# Evaluation of the effects of sedation administered via three different routes on the procedure, child and parent satisfaction during cystometry

**DOI:** 10.1186/s40064-016-3164-7

**Published:** 2016-09-06

**Authors:** Sengül Özmert, Feyza Sever, Hüseyin Tuğrul Tiryaki

**Affiliations:** 1 Department of Anesthesia, Ankara Childrens’ Health and Diseases Hematology and Oncology Training and Research Hospital, Kurtdereli Sok, 06110 Ankara, Turkey; 2 Department of Pediatric Urology, Ankara Childrens’ Health and Diseases Hematology and Oncology Training and Research Hospital, Ankara, Turkey

**Keywords:** Child, Drugs, Outpatient

## Abstract

**Purpose:**

In this study, we retrospectively investigated case reports with and without midazolam administration via oral, intranasal and rectal before cystometry procedure. We aimed to compare the data to evaluate the effects of sedation before cystometry on the pediatric patients and parents’ satisfaction.

**Methods:**

A total of 124 ASA I-II pediatric cases aged 5–14 years were retrospectively investigated from the hospital records. One of the three administration routes was chosen; oral midazolam at a dose of 0.5 mg/kg and nasal or rectal midazolam at a dose of 0.3 mg/kg (maximum 15 mg). Heart rate, blood pressure, oxygen saturation, the Wisconsin Hospital of Children Sedation Scale (CHWSS) score and the Groningen Distress Rating Scale (GDRS) score were recorded. Cystometry measurement values, diagnoses of the cases and procedure durations were recorded from the urodynamic laboratory records.

**Results:**

80 female, 44 male cases were evaluated. The CHWSS score at the 10th and 20th minutes after the drug administeration was higher in the oral group than the others (p = 0.001). The duration between the administration of the drug and the start of the procedure was shorter in the nasal group (p = 0.01). Parents satisfaction for sedation was 77 % when comparison of the cystometry with and without sedation. Comparison of the cystometry results with or without sedation no significant difference was found between all parameters (p > 0.01).

**Conclusion:**

We believe that sedation with midazolam administered through all three routes is a safe, effective and convenient option during cystometry, especially in the young age group.

## Background

Cystometry is an invasive investigation method used for the diagnosis and follow-up of urination disorders in children. Anxiety at a high rate of 61–71 % is known to develop during cystometry, originating from the unpleasant experiences of bladder and rectal catheterization (Phillips et al. 1996; Herd et al. 2006). Additionally, the request made by a stranger to fill the bladder and urinate in an unfamiliar environment increases the stress of the child and parents (Akil et al. 2005). These factors make cystometry difficult and can affect the result. The child should be able to stay calm and cooperate and the drug used for sedation should not affect the cystometry values so that an accurate and reproducible procedure can be ensured (Thevaraja et al. 2013).

Some studies report that the conscious sedation created by midazolam is appropriate and effective for many procedures and does not affect urodynamic measurements (Bozkurt et al. 1996; Ozkurkcugil and Ozkan 2010). Conscious sedation can be defined as a constant sedation in an awake patient where protective airway reflexes are maintained. Midazolam is a benzodiazepine with sedative, anxiolytic and amnestic effects. It is a valuable drug as it enables the patient to stay awake and cooperate for cystometry, has a rapid onset of action and short duration of effect without any serious side effects, causes partial anterograde amnesia, can be administered easily, and has several administration routes. The ability to reverse midazolam’s effect with flumazenil is a distinct advantage regarding safe use. This sedative agent can be administered through the intramuscular, intravenous, intranasal, rectal, oral or sublingual routes. Each administration route has specific advantages and disadvantages. Midazolam is effective when administered via the oral, nasal, rectal or sublingual routes (Akil et al. 2005; Malinovsky et al. 1995; Herd et al. 2006; Kogan et al. 2002). Its nasal or oral use can be difficult in children due to nasal irritation it causes and the unpleasant taste leading to spitting the drug out. Although the full dose of the drug can be made available with rectal administration, there may be fear and shame when this route is used.

Only a few studies have evaluated the effect of midazolam on children undergoing cystometry with and without sedation, the parents and the procedure itself. We aimed to investigate the effects of three different route of midazolam sedation on the patient who undergone cystometry retrospectively. And also effects of sedation on parents and the urodynamic procedure comparing the cases that had received and not received sedation before the cystometry procedure at our clinic.

## Methods

A total of 124 ASA I-II pediatric cases aged 5–14 years who underwent cystometry with or without sedation at the Pediatric Urology Clinic between 01 January 2014 and 31 August 2014 were retrospectively investigated from the hospital records. Ethics Committee permission dated 22.05.2014 and no. 2014-036 was obtained from the Ankara Child Health and Diseases, Hematology and Oncology Training and Research Hospital. Patients who underwent cystometry due to urinary incontinence, dysfunctional elimination syndrome, vesicoureteral reflux, past posterior urethral valve surgery, and recurrent urinary tract infection were included in the study and groups containing an equal number of subjects were created according to sedation status. Cases that were ASA III or ASA IV, and patients with an active urinary system infection, severe cardiac and respiratory dysfunction, airway abnormalities, known psychiatric disease, anxiolytic or sedative drug use, and a disease affecting urinary tract sensation (neurogenic bladder) were excluded from the study.

Sedation is not administered routinely for cystometry at the urodynamics outpatients of our hospital. The experienced urodynamics nurse assess all patients for sedative necessity. The cases that were to be sedated were fasted for minimum of 6 h before the procedure using the routine protocol of our Anesthesia Clinic. One of the three administration routes was chosen by the anesthetist preference: oral midazolam at a dose of 0.5 mg/kg and nasal or rectal midazolam at a dose of 0.3 mg/kg (maximum 15 mg) (9). The calculated dose was sprayed to the patient’s mouth through the syringe without dilution and the patient was instructed not to spit. The drug was administered with the apparatus named Intranasal Mucosal Atomization Device (LMA MAD Nasal, Teleflex, NC USA) with a spray mechanism that is fully inserted into the nostril and prevents leakage of the drug during nasal administration. For the rectal route, the 4–5 cm section of a feeding tube was cut and inserted on an injector tip. A lubricant gel was administered for ease of application. Midazolam was given through all three routes without being mixed with any other substance as it could change the concentration and effectiveness.

All patients had their parents with them during the procedure and the experienced urodynamics nurse administered a lubricating gel during urethral and rectal catheterization. We formed four groups with the same number of patients as oral, nasal, rectal and the control group not administered sedation and chose the patients for each group according to the order of the presentation date to the clinic. All patients were routinely monitorized at our clinic and heart rate, blood pressure, oxygen saturation, the Wisconsin Hospital of Children Sedation Scale (CHWSS) score (Table 1) (Thevaraja et al. 2013) and the Groningen Distress Rating Scale (GDRS) score (1 = Calm; 2 = Moderate distress; 3 = Severe stress, under control; 4 = Severe stress, out of control; 5 = Panic) (Herd et al. 2006) were recorded at room entry, the 10th and 20th minutes after drug administration, and at the catheterization, bladder filling, urination and room exit stages. Urethral and rectal catheter insertion during the cystometry procedure is allowed when the CHWSS score is 5 or less. Intravenous midazolam at a dose of 0.05 mg/kg was administered via a vascular access for sedation to patients with a GDRS value of 4 and above. The patients were sent home after being monitored at the recovery unit following the procedure. Cases with a GDRS score of 4 and higher who did not want sedation did not undergo cystometry as the results would not be healthy.

**Table 1:** Children’s Hospital of Wisconsin Sedation Scale (CHWSS)

Children’s Hospital of Wisconsin Sedation Scale (CHWSS)	
6	Anxious, agitated, has pain
5	Spontaneously awake without stimulation
4	Sleepy, eyes open or can be awakened easily with audible stimulation
3	Can be awakened with loud sound and light tactile stimulation
2	Can be awakened slowly (with difficulty) with constant painful stimulation
1	Sleepy and unresponsive to painful stimulation
0	Completely unresponsive to painful stimulation

The anesthesia forms, cystometry measurement values and diagnoses of the cases included in the evaluation were recorded from the hospital records. The duration from the administration of the drug until the procedure and the duration from the beginning of the procedure to the end were calculated from the urodynamic laboratory records. The families of the patients were called by phone and asked standard questionnaire at post procedure period whether they were satisfied by the sedation during the cystometry procedure by authors (SÖ, FS). The parents were also asked to compare the procedure with previous experiences, if any.

### Statistical analysis

Statistical analysis was performed by using IBM SPSS Statistics for Windows 22 (Armonk, NY: IBM Corp) software. Shapiro–wilk test was used to test normality assumption of numerical variables. Frequency, percentage for categorical data and mean ± SD or median (min–max) for numerical data were calculated as descriptive statistics. Chi square or Fisher’s exact test were used to compare categorical variables. Oneway ANOVA and posthoc Duncan test were used for normally distributed variables, Kruskal–Wallis test and posthoc Dunn test were used if the distribution is not normal for comparisions of groups. Wilcoxon test was used to compare the values before and after sedation. A p value of less than .01 was considered statistically significant.

## Results

The demographic data, duration between drug administration and the procedure, procedure duration, parental satisfaction and CHWSS values of the pediatric patients who received or did not receive sedation with midazolam through three different routes during cystometry at the Pediatric Urology Urodynamic Outpatients Department of our hospital were recorded with a retrospective analysis as shown in Table 2. No statistically significant difference was found between the groups in terms of gender, weight, ASA values and parental satisfaction.

**Table 2:** Demographic data, procedure durations and CHWSS values

	ORAL (n = 31)	RECTAL (n = 31)	NASAL (n = 31)	CONTROL (n = 31)	p value
Age	7.8 ± 2.5	8.1 ± 2.6	7.3 ± 2	8.4 ± 3.2	0.063
Female/male	18/13	23/8	19/12	20/11	0.578
Weight	27.2 ± 10.8	27 ± 8.3	26.9 ± 8.6	31.5 ± 13.8	0.258
ASA I/II	23/8	20/11	26/5	–	0.220
Duration between drug administration and the procedure	22.3 ± 2.3	21.7 ± 2	12.1 ± 2	–	0.000
Procedure duration	15.9 ± 7.3	14.4 ± 3.8	14.8 ± 7.3	20.7 ± 6.8	0.001
Parental satisfaction (very satisfied/satisfied/not satisfied)	24/7/0	18/13/0	23/8/0	22/8/1	0.380
10th min CHWSS Score	5 (4–6)	4 (3–6)	4 (4–6)	–	0.001
20th min CHWSS Score	4 (4–5)	4 (3–5)	–	–	0.001

The values were provided as mean ± SD, median (min–max) and numbers

There are no significant difference between mean age of the groups. The CHWSS score evaluated at the 10th and 20th minutes after the drug was administered was higher in the oral group than the others (p < 0.01). The duration between the administration of the drug and the start of the procedure was shorter in the nasal group (p < 0.01). All these results were statistically significant.

Urodynamic diagnoses in the groups with and without sedation are presented at Table 3.

**Table 3:** Urodynamic diagnoses in the groups with and without sedation

Urodynamic diagnosis	The group with sedation n = 93 (75 %)	The group without sedation n = 31 (25 %)
Normal urodynamic findings	31 (33 %)	8 (25 %)
Overactive bladder	21 (23 %)	2 (6 %)
Underactive bladder	3 (3 %)	5 (16 %)
Low capacity bladder	27 (29 %)	10 (33 %)
Dysfunctional urination	11 (12 %)	6 (20 %)

The hemodynamic data revealed higher blood pressure values, especially at catheterization and the later stages, in the control group. Following midazolam administration, two patients in the rectal group complained of double vision at the 20th minute while one patient in the rectal group and one in the oral group had hypotension (defined as a reduction of more than 20 % compared to the initial value) during the bladder filling stage but no statistically significant difference was found between the groups. None of the patients were found to have developed desaturation. None of the patients spitted during oral administration. Nasal burning was reported in 77 % of the patients during intranasal administration. The cystometry procedure is allowed when the CHWSS score is 5 or less in all group.

No statistically significant difference was present between the four groups in terms of GDRS scores at the room entry and cystometry stages. The values are presented at Table 4. No additional dose was required for GDRS increase in the three sedation groups.

**Table 4:** Groningen Distress Rating Scale (GDRS)

GDRS	ORAL (n = 31)	RECTAL (n = 31)	NASAL (n = 31)	CONTROL (n = 31)	p value
Room entry	1 (1–3)	1 (1–5)	1 (1–4)	1 (1–3)	0.845
Bladder catheterization	2 (1–3)	2 (1–3)	2 (1–3)	2 (1–3)	0.922
Rectal catheterization	1 (1–3)	2 (1–3)	2 (1–3)	2 (1–3)	0.508
Bladder filling	1 (1–2)	1 (1–3)	1 (1–3)	1 (1–2)	0.102
Urination	1 (1–2)	1 (1–3)	1 (1–3)	1 (1–3)	0.868
Room exit	1 (1–2)	1 (1–3)	1 (1–2)	1 (1–3)	0.499

The values are presented as median (min–max)

Thirteen of the patients who received sedation had undergone a cystometry procedure without sedation about a year ago. The cystometry values of these patients before and after sedation are compared in Table 5. The parents of the same patients were asked to compare the two procedures regarding sedation. Ten of the 13 parents expressed that the procedure under sedation was better while 2 stated no difference between the two and 1 stated that the procedure without sedation was better. Prior urethral catheterizations were performed in seven patients of study group. These patient’s parents also are satisfied for sedation.

**Table 5:** The cystometry values of 13 patients with and without sedation

	Without sedation	With sedation	p value
Uroflow results			
Peakflowrate (ml/s)	13	26	0.056
Voided volume (ml)	131	179	0.463
Voided time (s)	19	16	0.184
Average flowrate (ml/s)	6	14	0.029
Filling phase results			
İnfused volume (ml)	166	222	0.506
Max. vesical pressure (cmH_2_O)	62	58	0.917
Sensation results			
Bladder filling			
First sensation (ml)	57	67	0.197
Normal desire (ml)	87.5	94	0.155
Strong desire (ml)	127	106	0.350
Max cyst cap. (ml)	148.5	181.5	0.192
Detrusor pressure			
First sensation (cmH_2_O)	10.5	8.5	0.755
Normal desire (cmH_2_O)	18	13	0.688
Strong desire (cmH_2_O)	25	26.5	0.625
Max cyst cap. (cmH_2_O)	27	34.5	0.214
Compliance results (linear regression)			
Pves compliance (ml/cmH_2_O)	7.75	6.60	0.753
Pdet compliance (ml/cmH_2_O)	5.05	5.05	0.588
Voiding phase results			
Max. detrusor pressure (cmH_2_O)	57	81	0.465

## Discussion

Urodynamic studies are the basic diagnostic method in children with a lower urinary tract symptom and the procedures may need to be repeated to monitor the effectiveness of the treatment. The bladder and rectal catheterization performed during this procedure cause fear and anxiety in pediatric patients. Children may show extreme anxiety during this procedure, especially if they have previous painful experiences. It is therefore sometimes not possible to perform an urodynamic study in these patients. Prevention of stress is required to eliminate the effects of previous painful experiences in children. Methods such as having the parents present, the use of lidocaine gel during catheterization, distraction techniques, warm infusion fluids and hypnosis have been recommended because they decrease the pain and anxiety that occur due to the catheterization during the procedure (Gray 1996; Goodman et al. 2003; Gerard et al. 2003; Butler et al. 2005). However, some children continue to show serious stress despite these methods and sedation becomes inevitable in such cases. Various anesthetic agents such as propofol, ketamine, midazolam, opioids, and inhalation anesthetics have been used for this purpose. However, some of these drugs are known to affect cystometry results. A pediatric urodynamics study conducted under general anesthesia with sevoflurane and nitric oxide concluded that the external urethral sphincter function can be evaluated under general anesthesia but the detrusor reflex cannot be evaluated as it is suppressed (Ameda et al. 1997). Malinovski et al. reported from their study investigating the effects of intravenous opioids and ketoprofen on urodynamic parameters in male adults that opioids change urodynamic results in contrast to ketoprofen but the opioid nalbuphine has no effect on detrusor contraction (Malinovsky et al. 1998). Ceran et al. reported that midazolam depressed detrusor contraction at moderate and high concentrations in the study that they conducted to determine the effects of the commonly used anesthetic agents of ketamine, propofol and midazolam on rat detrusor smooth muscle in vitro (Ceran et al. 2010). Another study investigated the effects of ketamine, propofol, midazolam and ether on cystometric parameters in rats and reported that the urination reflex disappears under ketamine anesthesia while propofol prolongs the urination period but ether inhalation and midazolam make no change in urodynamic parameters (Ozkurkcugil and Ozkan 2010).

Benzodiazepines provide sedation, anxiolysis and amnesia but do not have an analgesic effect. Midazolam is a benzodiazepine with a rapid effect of short duration and enables quick recovery. It provides great flexibility in terms of drug administration options, especially in pediatric cases, since it can be administered via various routes such as oral, intranasal, sublingual, rectal, intramuscular and intravenous. Oral administration is highly preferred for sedation in children because it does not cause pain and stress. However, its bad taste sometimes makes oral use of the agent difficult. Only the ampoule form is available in our country and sometimes the required dose cannot be given this way due to spitting or vomiting of the patient. The nasal administration of midazolam provides fast and effective sedation due to the rich vascular supply and large mucosal surface but usually causes irritation and burning. It is advantageous as it enables achieving the desired sedation level with a lower dose and earlier and also completing the intervention quicker. The rectal route enables the intended dose to be fully received by the patient and eliminates risks such spitting by the patient and nasal stinging, making it a reliable alternative route. A disadvantage of this method is the possible shame or discomfort of the child during the procedure. A sufficient sedative and anxiolytic effect was seen in all groups in a study where the effectiveness of midazolam administered through four different routes (oral, nasal, rectal and sublingual) as premedication was investigated (Kogan et al. 2002). Midazolam is administered orally, nasally or rectally to children for sedation in the urodynamics outpatient department of our hospital as it is not invasive. The administration of midazolam through any of the three different routes was seen to provide safe and adequate sedation in our study. The higher CHWSS scores in the oral administration group suggest lower effectiveness of sedation in this group. We think lower absorbion rate than nasal group and spitting and vomiting of the patients because of bad taste cause lower effectiveness of oral midazolam sedation group. Because of retrospective construction of our study,we did not measure plasma concentration of three different administration of midazolam. However, no significant difference was found between the four groups regarding GDRS during the cystometry process.

Although there are no statistically difference between mean age of the groups, the control group’s mean age was seen a bit higher (8.4 ± 3.2) than the others in our study. The reason for the unwillingness in the control group that did not request sedation and the lack of a difference in the GDRS values could be the high mean age of the patients in this group and therefore the easier acceptance of the cystometry procedure. Sweeney et al. found the mean age in the group that did not require sedation to be significantly higher (8.1 ± 5.1 years) and concluded that children aged 3–7 years should be assessed in terms of sedation in their retrospective study on the need for sedation during urodynamic investigations in children (Sweeney et al. 2008). Sedation is not administered routinely for cystometry at the urodynamics outpatients of our hospital. The experienced urodynamics nurse assess all patients for sedative necessity. Therefore, we can decrease the number of unsuccessful, incomplete or failed procedures with the early detection of patients who are anxious and have difficulty complying and are believed to require sedation by the experienced urodynamics nurse.

The agent used for sedation in children should enable performing the procedure by decreasing stress and should also not affect the measurements. Bozkurt et al. reported that a single dose of intranasal midazolam did not affect the urodynamic results and provided adequate comfort for the procedure in their study investigating the effect of intranasal midazolam on lower urinary system function in children undergoing an urodynamic study (Bozkurt et al. 1996). The Frankl Behavior Rating Scale (FBRS) scores during urinary and rectal catheterization in children administered a low dose of ketamine or midazolam were better in the ketamine group. Nine patients in the ketamine group and 12 patients in the midazolam group had undergone an urodynamic procedure without sedation in the past and no difference was reported between the two groups when the old and new data of the patients were compared in the same study. Family satisfaction was good in both groups (Thevaraja et al. 2013). Akil et al. concluded that sedation with midazolam is effective in children undergoing voiding cystourethrogram (VCUG) and has no negative effect on the results of the procedure in a study where they compared midazolam and chloral hydrate (Akil et al. 2005). The rates of parental satisfaction were high in all three sedation groups in our study. Thirteen patients who received sedation in this study had undergone cystometry without sedation about a year ago. Also seven children who received sedation had undergone VCUG and urethral catheterization without sedation about six months ago. The parents of these children were asked to compare the procedures regarding sedation administration and only one parent felt that the non-sedated procedure was better while two parents said that there was no difference and all the other parents stated that they preferred sedation. Comparison of the cystometry results with or without sedation in this patient group revealed no significant difference was found between all parameters (p > 0.01). Although the studies were done a year apart, the lack of a difference between the results indicates that midazolam generally has no effect on cystometry results.

The limitations of our study retrospective course, small sample size, selection bias.

## Conclusion

The child needs to be calm and cooperating during the invasive procedure of cystometry. Effective sedation was found with all three administration routes but we feel that the quicker onset of the sedation and lower dose used with the nasal route compared to the oral group are advantages despite the nasal stinging. An advantage of sedation was that cystometry was performed in a shorter time in the sedated patients compared to the control group. We believe that sedation with midazolam administered through all three routes is a safe, effective and convenient option during cystometry, especially in the young age group. Most of the parents are pleased with sedation application.

## Abbreviations

ASA: American Society of Anesthesiologists
CHWSS: Wisconsin Hospital of Children Sedation Scale
GDRS: Groningen Distress Rating Scale
VCUG: voiding cystourethrogram

## References

[CR1] Akil I, Ozkol M, YilmazIkizoglu O (2005). Premedication during micturating cystourethrogram to achieve sedation and anxiolysis. Pediatr Nephrol.

[CR2] Ameda K, Kakizaki H, Yamashita T (1997). Feasibility of urodynamic study (combined cystometry and electromyography of the external urethral sphincter) under general anesthesia in children. Int J Urol.

[CR3] Bozkurt P, Kilic N, Kaya G (1996). The effects of intranasal midazolam on urodynamic studies in children. Br J Urol.

[CR4] Butler LD, Symons BK, Henderson SL (2005). Hypnosis reduces distress and duration of an invasive medical procedure for children. Pediatrics.

[CR5] Ceran C, Pampal A, Goktaş O (2010). Commonly used intravenous anesthetics decrease bladder contractility: an in vitro study of the effects of propofol, ketamine and midazolam on the rat bladder. Indian J Urol.

[CR6] Gerard LL, Cooper CS, Duethman KS (2003). Effectiveness of lidocaine lubricant for discomfort during pediatric urethral catheterization. J Urol.

[CR7] Goodman TR, Kilborn T, Pearce R (2003). Warm or cold contrast medium in the micturatingcystourethrogram (MCUG): which is the best?. Clin Radiol.

[CR8] Gray M (1996). Traumatic urethral catheterization of children. Pediatr Nurs.

[CR9] Herd DW, McAnulty KA, Keene NA (2006). Conscious sedation reduces distress in children undergoing voiding cystourethrography and does not interfere with the diagnosis of vesicoureteric reflux: a randomized controlled study. Am J Roentgenol.

[CR10] Herd DW, McAnulty KA, Keene NA (2006). Conscious sedation reduces distress in children undergoing voiding cystourethrography and does not interfere with the diagnosis of vesicoureteric reflux: a randomized controlled study. AJR.

[CR11] Kogan A, Katz J, Efrat R (2002). Premedication with midazolam in young children: a comparison of four routes of administration. Paediatr Anaesth.

[CR12] Malinovsky JM, Populaire C, Cozian A (1995). Premedication with midazolam in children. Effect of intranasal, rectal and oral routes on plasma midazolam concentrations. Anaesthesia.

[CR13] Malinovsky JM, LeNormand L, Lepage JY (1998). The urodynamic effects of intravenous opioids and ketoprofen in humans. Anesth Analg.

[CR14] Ozkurkcugil C, Ozkan L (2010). Effects of anesthetics on cystometric parameters in female rats. Int Urol Nephrol.

[CR15] Phillips D, Watson AR, Collier J (1996). Distress and radiological investigations of the urinary tract in children. Eur J Pediatr.

[CR16] Sweeney H, Rzepski B, Hochman H (2008). Identifying characteristics of children requiring sedation for urodynamics. Urol Nurs.

[CR17] Thevaraja AK, Batra YK, Rakesh SV (2013). Comparison of low-dose ketamine to midazolam for sedation during pediatric urodynamic study. Pediatr Anesth.

